# Effects of blunt force injuries in third-instar *Drosophila* larvae persist through metamorphosis and reduce adult lifespan

**DOI:** 10.17912/micropub.biology.000423

**Published:** 2021-07-13

**Authors:** Jorgo Lika, Rebeccah J Katzenberger, Barry Ganetzky, David A Wassarman

**Affiliations:** 1 Department of Medical Genetics, School of Medicine and Public Health, University of Wisconsin-Madison, Madison, WI 53706; 2 Department of Genetics, College of Agricultural and Life Sciences, University of Wisconsin-Madison, Madison, WI 53706

## Abstract

Blunt force injuries are a significant cause of disability and death worldwide. Here, we describe a *Drosophila melanogaster* model of blunt force injury that can be used to investigate cellular and molecular mechanisms that underlie the short-term and long-term effects of injuries sustained at a juvenile stage of development. Injuries inflicted in late third-instar larvae using the spring-based High-Impact Trauma (HIT) device robustly activated the humoral defense response process of melanization and caused larval and pupal lethality. Additionally, adults that developed from injured larvae had reduced lifespans, indicating that cellular and molecular mechanisms activated by blunt force injuries in larvae persist through metamorphosis and adult development. Previously, the HIT device has been used to investigate genetic and environmental factors underlying mechanisms that contribute to consequences of blunt force injuries incurred in adult flies. This work expands use of the HIT device to a juvenile stage of development, offering the opportunity to investigate whether the consequences of blunt force injuries involve different factors and mechanisms at different stages of development.

**Figure 1. Blunt force injuries in late third-instar larvae cause extensive melanization and reduce adult lifespan:**
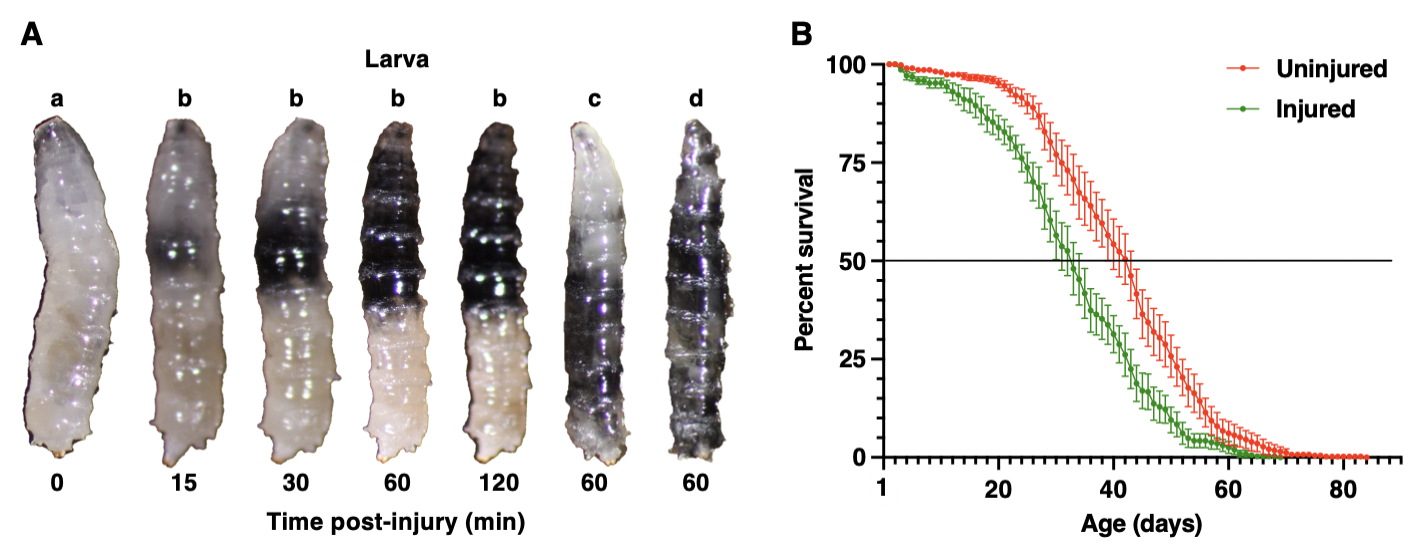
(A) Representative images of four different *w^1118^* third-instar larvae (a-d) at the indicated times after blunt force injuries from the HIT device. Larva b was imaged at 15, 30, 60, and 120 min. Black regions are melanized. Anterior is at the top. (B) Percent survival vs. age for adult flies either injured (n=257 total flies) or not injured (n=460 total flies) by the HIT device as third-instar larvae. The horizontal line at 50% indicates the median lifespan. Error bars indicate the standard error of the mean for at least three independent trials.

## Description

Blunt force injuries are non-penetrating injuries to the body that result from powerful impacts with dull objects, as commonly occurs in motor vehicle crashes, contact sports, and falls. Blunt impacts generate a combination of forces, including acceleration and deceleration, shearing, and crushing forces, that directly injure tissues and can lead to long-term detrimental consequences. For example, blunt force injuries to the brain, also known as traumatic brain injury (TBI), can lead to progressive brain atrophy and increased susceptibility to neurodegenerative disorders (Johnson *et al.* 2017).

To investigate both the immediate effects of blunt force injury at a juvenile stage in *Drosophila* as well as its long-term consequences at later developmental stages, we used the High-Impact Trauma (HIT) device to inflict blunt force injuries in third-instar larvae (Katzenberger *et al.* 2013). *Drosophila* have three distinct stages of post-embryonic development, larva (three instar stages), pupa, and adult. During metamorphosis in the pupal stage, most larval tissues are degraded and many new adult-specific tissues and structures are formed. For example, much of the larval nervous system is reorganized. Larval sensory neurons degenerate and are replaced by newly arising adult neurons (Truman 1990; Tissot and Stocker 2000). In addition, some adult interneurons and the vast majority of adult motor neurons are remodeled from larval neurons. The substantial remodeling that occurs during metamorphosis raises the question of whether effects of blunt force injuries delivered to larvae persist through metamorphosis and have detrimental consequences in adults. If so, *Drosophila* could serve as a useful model to investigate mechanisms by which the juvenile occurrence of blunt force injuries, including TBI, in humans can result in long-term detrimental effects in adults.

To inflict blunt force injuries in larvae, we used forceps to place late (wandering) third-instar *w^1118^* larvae at the bottom of otherwise empty plastic fly vials, and we subjected vials of 60 larvae to four strikes from the HIT device with 5 min between strikes. The HIT device consists of a metal spring clamped at one end to a board. A fly vial is connected to the free end of the spring and positioned over a polyurethane pad. Bending and release of the spring causes the vial to rapidly contact the pad. By marking the original location of larvae in a vial, we found that larvae, which naturally adhere to the vial wall, were dislodged when the vial contacted the pad. Thus, strikes from the HIT device presumably led to blunt force injuries when the vial contacted the pad and when airborne larvae struck the vial wall. We transferred injured larvae in groups of 20 to vials containing cornmeal-molasses food and incubated them at 25^o^C. Control larvae were treated similarly except not subjected to strikes from the HIT device.

We examined individual larvae by eye and found that 30.3% of injured larvae (n=420), but only 0.7% of uninjured larvae (n=420), began turning black about 15 min after the injury (t-test, *p*<0.05) and that blackening continued to spread from initiation sites for about 2 h (compare larvae a and b in Fig. 1A). The location of blackening varied among larvae. Some larvae had small areas of blackening while others had blackening that covered the anterior or posterior half of the larva or the whole larva (larvae b, c, and d, respectively, in Fig. 1A). Blackening is likely due to melanization, an innate immune reaction at the site of wounding that serves to contain microbial pathogens and facilitate wound healing (Tang 2009). Polymerized melanin is produced by oxidation of phenols to quinones by phenoloxidase, which is activated by cleavage of prophenoloxidase in response to innate immunity signals. Melanization can be induced by pricking larvae with a fine needle, but in this case, blackening is confined to a small area directly surrounding the wound site (Binggeli *et al.* 2014), indicating either that the HIT device injures large regions of larvae or that the melanization reaction spreads from the site of injury.

Blunt force injuries in third-instar larvae caused mortality at each stage of development. 8.5% of uninjured larvae (n=420) and 63.2% of injured larvae (n=420) died as larvae (t-test, *p*<0.05). All of the 30.3% of injured larvae that melanized as larvae died as larvae, and none of the remaining 32.9% of injured larvae that died as larvae melanized. In addition, 4.3% of uninjured and 13.6% of injured larvae died as pupae (t-test, *p*<0.05). The remaining 87.2% of uninjured larvae and 23.2% of injured larvae survived to the adult stage. While pricking of adult flies causes melanization (Scherfer *et al.* 2008), we did not observe melanization in the adults that developed from injured larvae, indicating that the melanization reaction induced in larvae does not persist through metamorphosis. In contrast, relative to adults that developed from uninjured larvae, adults that developed from injured larvae had significantly reduced lifespans, as assessed by the Kaplan-Meier Fisher’s Exact Test (*p*=6.6X10^-10^ at 25%, *p*=9.9X10^-10^ at 50%, *p*=1.1X10^-7^ at 75%) (Fig. 1B). The median lifespan of adult flies following larval injury was reduced from 42.3 to 32.4 days, and their maximum lifespan was reduced from 84 to 69 days. These data suggest that blunt force injuries to larvae activate cellular and molecular mechanisms that possibly accelerate aging.

The goal of this work was to establish a *Drosophila* model for investigating the short-term and long-term consequences of blunt force injuries occurring at juvenile stages of development. We found that blunt force injuries to third-instar larvae by the HIT device resulted in rapid and extensive melanization of larvae, death of larvae and pupae, and reduced lifespan of adults. Because of the experimental tractability of *Drosophila*, the blunt force injury model offers considerable potential for understanding the cellular and molecular mechanisms that underlie the long-term physiological consequences of blunt force injuries, including neurodegeneration in TBI. For example, application of the HIT device to larvae was used to investigate the influence of TBI in progression of neurological disorders (Anderson *et al.* 2018, 2021) Additionally, the model provides the opportunity to determine whether blunt force injuries in larvae and adults reduce adult lifespan by the same or different mechanisms. The model may also provide insights into other types of environmental trauma to third-instar larvae that reduce adult lifespan, including irradiation, sublethal cold, and hypoxia (Sudmeier *et al.* 2015; Koštál *et al.* 2019; Polan *et al.* 2020).

## Methods

***Blunt force injury of larvae using the HIT device***

*w^1118^* flies were obtained from Dr. Gerald Rubin’s lab (University of California-Berkeley) and maintained for 25 years. Fly development was synchronized by culturing adult *w^1118^* flies on molasses-agar egg-laying plates (Bai *et al.* 2009) with yeast paste for 2 h at 25^o^C. Plates were then incubated for 4 days at 25^o^C, at which point the hatched larvae had developed to the wandering third-instar stage. Larvae were transferred to the bottom of empty plastic fly vials using Dumont #5 forceps (Fine Science Tools), and the movement of larvae was restricted to the bottom 1 inch of vials by plugging vials with a cotton ball covered with plastic wrap, which prevented larvae from sticking to the cotton. Vials containing 60 larvae received four strikes from the HIT device with 5 min between strikes, as described in Katzenberger *et al.* (2013, 2015a). Larvae were then transferred in groups of 20 to vials containing cornmeal-molasses food (Katzenberger *et al.* 2015b) and incubated at 25^o^C. Percent melanization and larval and pupal lethality were determined by visual inspection of 21 vials of 20 larvae (n=420). Larvae were considered dead if they did not show obvious locomotor activity, and pupae were considered dead if they did not eclose as adults.

***Lifespan assay***

Adult flies were collected at 1 day post-eclosion and placed in vials with 20 flies each (approximately 10 males and 10 females). The number of surviving flies was counted daily until all flies had died. Flies were transferred to new vials approximately every 3 days, and they were not combined when the number of flies was low. Flies were considered dead if they did not show obvious locomotor activity. Statistical analysis of survival by the Kaplan-Meier Fisher’s Exact Test was performed using OASIS 2 (Online Application for Survival Analysis 2) (Han *et al.* 2016).
